# Characterization of the axon initial segment (AIS) of motor neurons and identification of a para-AIS and a juxtapara-AIS, organized by protein 4.1B

**DOI:** 10.1186/1741-7007-9-66

**Published:** 2011-09-29

**Authors:** Amandine Duflocq, Fabrice Chareyre, Marco Giovannini, François Couraud, Marc Davenne

**Affiliations:** 1 INSERM UMRS 952, 9 Quai St Bernard, F-75005, Paris, France; 2 CNRS UMR 7224, 9 Quai St Bernard, F-75005, Paris, France; 3 UPMC Univ Paris 06, F-75005, Paris, France; 4 Division of Clinical and Translational Research, House Research Institute, Los Angeles, CA 90057, USA

## Abstract

**Background:**

The axon initial segment (AIS) plays a crucial role: it is the site where neurons initiate their electrical outputs. Its composition in terms of voltage-gated sodium (Nav) and voltage-gated potassium (Kv) channels, as well as its length and localization determine the neuron's spiking properties. Some neurons are able to modulate their AIS length or distance from the soma in order to adapt their excitability properties to their activity level. It is therefore crucial to characterize all these parameters and determine where the myelin sheath begins in order to assess a neuron's excitability properties and ability to display such plasticity mechanisms. If the myelin sheath starts immediately after the AIS, another question then arises as to how would the axon be organized at its first myelin attachment site; since AISs are different from nodes of Ranvier, would this particular axonal region resemble a hemi-node of Ranvier?

**Results:**

We have characterized the AIS of mouse somatic motor neurons. In addition to constant determinants of excitability properties, we found heterogeneities, in terms of AIS localization and Nav composition. We also identified in all α motor neurons a hemi-node-type organization, with a contactin-associated protein (Caspr)^+ ^paranode-type, as well as a Caspr2^+ ^and Kv1^+ ^juxtaparanode-type compartment, referred to as a para-AIS and a juxtapara (JXP)-AIS, adjacent to the AIS, where the myelin sheath begins. We found that Kv1 channels appear in the AIS, para-AIS and JXP-AIS concomitantly with myelination and are progressively excluded from the para-AIS. Their expression in the AIS and JXP-AIS is independent from transient axonal glycoprotein-1 (TAG-1)/Caspr2, in contrast to juxtaparanodes, and independent from PSD-93. Data from mice lacking the cytoskeletal linker protein 4.1B show that this protein is necessary to form the Caspr^+ ^para-AIS barrier, ensuring the compartmentalization of Kv1 channels and the segregation of the AIS, para-AIS and JXP-AIS.

**Conclusions:**

α Motor neurons have heterogeneous AISs, which underlie different spiking properties. However, they all have a para-AIS and a JXP-AIS contiguous to their AIS, where the myelin sheath begins, which might limit some AIS plasticity. Protein 4.1B plays a key role in ensuring the proper molecular compartmentalization of this hemi-node-type region.

## Background

**Figure 1:**
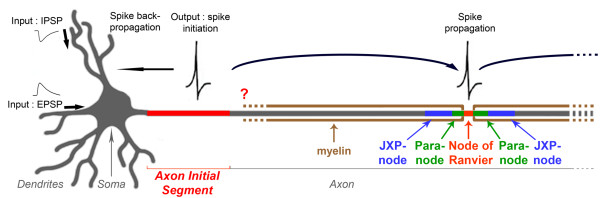
**Schematic drawing of a neuron showing the position of the axon initial segment (AIS)**. The AIS plays two crucial roles in neurons: it forms a barrier between the somatodendritic and axonal compartments maintaining the neuron's polarity, and the AIS is the site where electrical outputs are initiated: aggregation of specific voltage-gated ion channels allows spikes to be generated in response to inputs coming from the somatodendritic compartment. Once initiated, spikes are both propagated along the axon and retropropagated towards the soma and dendrites. When axons are myelinated, spikes are propagated in a saltatory fashion, from a node of Ranvier to the next one. What is the precise AIS ion channel composition? And does the myelin sheath start immediately after the AIS or, as a corollary, is the axon organized at the first myelin-anchoring site as a hemi-node of Ranvier, with a paranode-like and a juxtaparanode-like compartment? These questions are addressed in this study.

The ability of the nervous system to convey information relies on the ability of its neurons to translate the information they receive into electrical outputs that can be propagated to their target cells. This crucial property takes place in the AIS (Figure 1), and is due to the aggregation of voltage-gated sodium (Nav) and voltage-gated potassium (Kv) channels. Depending on the combination and distribution of Nav and Kv channel isoforms at the AIS, neurons are able to generate spikes with different shapes, frequencies and patterns [1]. Very recently, AIS length and distance from the soma have also been shown to modify a neuron's spiking properties and to be modulated by neural activity [2-4]. It is therefore crucial to characterize all these parameters and determine where the myelin sheath begins in order to assess a given neuron's excitability properties and its ability to display the latter AIS plasticity mechanisms. Yet, only some of these criteria have been addressed, independently and in different neuronal types. In addition, despite the crucial role of the AIS as the spike-generating region, the molecular and potential domain organization of the axon immediately following the AIS, which might have an important impact on the neuron's spiking properties, has never been studied. If the myelin sheath starts immediately after the AIS, thus abutting an AIS instead of a node of Ranvier, another question arises: how would the axon be organized in this region? Since AISs differ from nodes of Ranvier in terms of molecular composition (hence of molecular clustering mechanisms) and development (AISs are preorganized when myelination takes place), would this first myelin-anchoring region resemble a hemi-node of Ranvier: would the AIS be, like nodes of Ranvier, flanked by a paranode-like and a juxtaparanode-like compartment (Figure 1)?

Several studies have identified Nav and Kv isoforms that can be expressed specifically at the AIS: (i) Nav1.1, Nav1.2 and Nav1.6 [5-7], clustered at the AIS by ankyrin G (AnkG) [8,9], a cytoskeleton-linked scaffolding protein expressed throughout all AISs [10]; (ii) Kv1.1 and Kv1.2 [2,7,11-15], as well as Kv7.2/KCNQ2 and Kv7.3/KCNQ3 [16-18].

While AnkG also clusters KCNQ2/3 at the AIS [17], the mechanisms controlling expression of Kv1 channels at the AIS remain unresolved. PSD-93 was shown to play a role, although it may be compensated for *in vivo *[15,19]. Clustering of Kv1 channels has been mostly studied in juxtaparanodes, which are separated from nodes of Ranvier by paranodes: clustering of Kv1 channels requires the juxtaparanodal cell adhesion complex consisting of the axonal proteins transient axonal glycoprotein-1 (TAG-1) and contactin-associated protein-like 2 (Caspr2) [20-22], controlled by glial TAG-1 [23]. The contiguous Caspr-expressing paranode is also necessary: it forms a barrier, which excludes Kv1 channels from paranodes and confines them to juxtaparanodes [24-27]. The cytoskeletal linker protein, protein 4.1B, which binds both Caspr and Caspr2 [28], might play a key role in stabilizing their expression in paranodes and juxtaparanodes, respectively, thus ensuring the proper compartmentalization of Kv1 channels. However, 4.1B has been shown to be necessary in juxtaparanodes only, controlling the distribution of Caspr2 and thus the expression of Kv1 channels [29].

Somatic motor neurons (MNs) constitute a functionally heterogeneous population of neurons that can be subdivided into different functional subgroups, the most prominent division being between α and γ MNs [30]. Despite this functional heterogeneity, the above-mentioned AIS determinants of excitability properties have hardly been investigated [17,31].

We have analyzed the AIS of somatic MNs and identified heterogeneous as well as homogeneous determinants of AIS spiking properties. We also provide, to our knowledge, the first molecular characterization of the axonal region directly following the AIS. We identified a paranode-like compartment and a juxtaparanode-like compartment, which support the beginning of the myelin sheath immediately after the AIS, and that, given the differences between nodes of Ranvier and AISs, we refer to as a 'para-AIS' and a 'juxtapara-AIS'. We also demonstrate that Kv1 channels appear at the AIS, para-AIS, and juxtapara-AIS concomitantly with myelination, and are progressively excluded from the para-AIS. We found that expression of Kv1 channels in the AIS and JXP-AIS is independent from TAG-1/Caspr2, in contrast to juxtaparanodes, and from PSD-93. Finally, we demonstrate that protein 4.1B is necessary to form the Caspr-expressing para-AIS barrier and to ensure the proper compartmentalization of Kv1 channels as well as the segregation of the AIS, para-AIS and juxtapara-AIS.

## Results

### AIS localization and length in motor neurons

**Figure 2:**
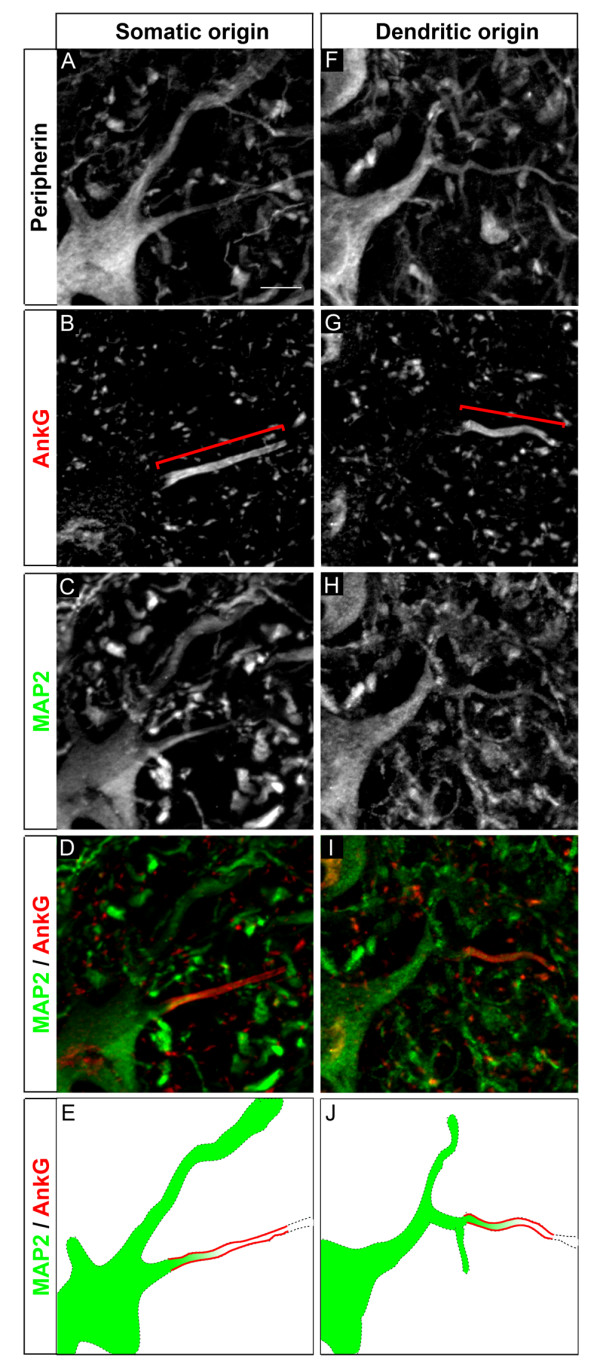
**Localization of axon initial segments (AISs) in motor neurons (MNs)**. Triple immunostaining of Peripherin **(A, F)**, ankyrin G (AnkG) **(B, G) **(brackets indicate the AIS) and microtubule-associated protein 2 (MAP2) **(C, H) **(AnkG and MAP2 are merged in **(D, I)**) in the adult mouse lumbar spinal cord showing two populations of MN AISs: emanating directly from the soma **(A-E) **or from a primary MAP2^+ ^dendrite **(F-J)**. **(E, J) **Simplified drawing of the merged image, respectively in (D) and (I). Note that the green MAP2 staining progressively decreases within the red AnkG^+ ^AIS. Scale bar = 10 μm.

We first analyzed the localization of the AIS in somatic MNs. For this we used an anti-Peripherin antibody, which in the spinal ventral horn specifically labels somatic MNs [32]. We used this antibody throughout our study instead of the more common MN marker, the anti-choline acetyltransferase (ChAT) antibody [33], not compatible with the low tissue fixation conditions (required for analyzing the expression of Nav and Kv channels: see below). We verified that all anti-ChAT labeled somatic MNs were also labeled by the anti-Peripherin antibody (data not shown). AISs were labeled with an antibody against AnkG. In the majority of MNs analyzed throughout the ventral horn of the lumbar spinal cord, the AIS was located on a neurite originating directly from the soma (Figure 2A-E), while other MNs had their AIS located on a secondary branch (Figure 2F-J). We used a somatodendritic marker, an antibody against microtubule-associated protein 2 (MAP2), to show that this secondary branch emanated from a dendrite (Figure 2F-J). Interestingly, even for AISs located on a branch that emanates directly from the soma (Figure 2A-E), MAP2 was expressed in their proximal portion, between the soma and the AnkG-labeled AIS, showing that the axon hillock has a somatodendritic identity. MAP2 staining progressively faded away at some distance within the AIS (Figure 2C, H), which reflects the barrier formed by the AIS between the somatodendritic and axonal compartments [34].

We analyzed whether this first heterogeneity regarding AIS origin correlated with different functional populations of MNs. We thus analyzed the distribution of these AISs in the two major types of MNs: α and γ. Interestingly, we found that α MNs (identified as Err3^-^/NeuN^+^; data not shown; [35]) are divided into two subpopulations: 15% of them had a dendrite-derived AIS, while the remaining 85% had a soma-derived AIS (Table 1). As for γ MNs (identified as Err3^+^/NeuN^-^; data not shown; [35]), all of them had their AIS originating directly from the soma (Table 1).

**Table 1:** Axon initial segment (AIS) origin, length, distance from the soma and voltage-gated sodium (Nav) channel composition in α and γ motor neurons (MNs)

	AIS origin	Length (μm)	Distance from the soma (μm)	Nav composition	
				
				Nav1.1/Nav1.6	Nav1.6
α-MN	Dendritic (15%) (*n *= 19)	29.5 ± 3.2 (*n *= 6)	16.9 ± 13.2 (*n *= 6)	100% (*n *= 18)	0% (*n *= 18)
	
	Somatic (85%) (*n *= 61)	29.3 ± 5.4 (*n *= 26)	5.6 ± 3.8 (*n *= 26)	79.8%, (*n *= 35)	20.2% (*n *= 35)

γ-MN	Somatic (100%) (*n *= 69)	28 ± 8.7 (*n *= 69)	8.1 ± 3.5 (*n *= 69)	81.2% (*n *= 43)	18.8% (*n *= 43)

We next measured the length of the AIS and its distance from the soma. We found that overall MN AISs had a very uniform length (30 ± 4.4 μm; *n *= 32; Table 1), which does not show statistically significant differences when comparing (for α MNs) dendrite-derived (29.5 ± 3.2 μm; *n *= 6) and soma-derived AISs (29.3 ± 5.4 μm; *n *= 26) or when comparing soma-derived AISs from α (29.3 ± 5.4 μm; *n *= 26) and γ MNs (28 μm ± 8.7; *n *= 69). The distance between the soma and the AIS showed a greater variability in particular for dendrite-derived AISs, whose distance from the soma was nonetheless longer in average (16.9 ± 13.2 μm; *n *= 6) than soma-derived AISs (for α MNs: 5.6 μm ± 3.8; *n *= 26; *P *< 0.05).

### Nav channel distribution in MN AISs

**Figure 3:**
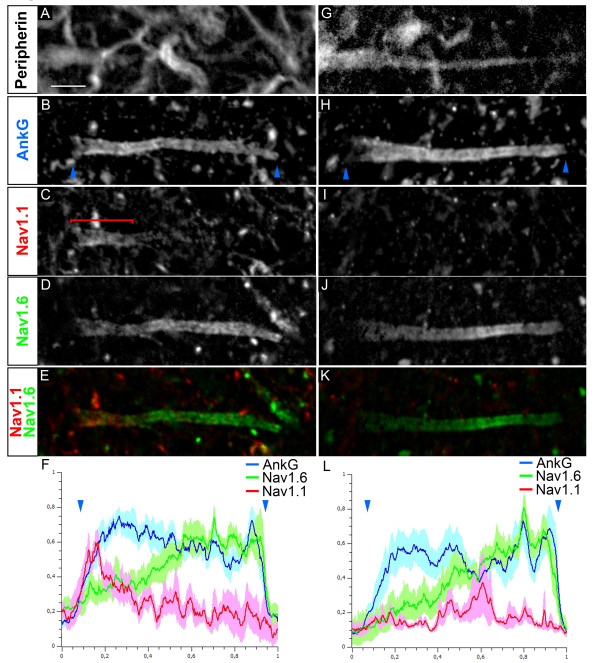
**Voltage-gated sodium (Nav) channel distribution in the axon initial segments (AISs) in motor neurons (MNs)**. Quadruple immunostaining of Peripherin **(A, G)**, ankyrin G (AnkG) **(B, H)**, Nav1.1 **(C, I) **and Nav1.6 **(D, J) **(Nav1.1 and Nav1.6 are merged in **(E, K)**) showing two populations of AISs: expressing Nav1.1 in a proximal compartment complementary to Nav1.6 expression **(A-F) **or expressing Nav1.6 alone throughout the AIS **(G-L)**. (F, L) The mean immunofluorescence intensity profile (shown by the line) ± SEM from *n *= 6 AISs is shown for AnkG, Nav1.1 and Nav1.6. For each AIS and each antibody, immunofluorescence intensities were normalized relative both to its maximum intensity along the AIS and to the length of the AIS. The beginning and the end of the AnkG^+ ^AIS in B and H are shown (also in F and L) by blue arrowheads. The bracket indicates the proximal AIS Nav1.1 expression domain. Scale bar = 5 μm.

In order to characterize the MN AIS excitability properties, we analyzed the AIS composition in terms of ion channels, and started with Nav channels. We investigated the expression of Nav1 channels in the AnkG^+ ^AIS of Peripherin^+ ^motor axons. We did not find any AIS expression of Nav1.2 in MNs (data not shown). We found that 79.8% of MNs (from *n *= 35) expressed both Nav1.1 and Nav1.6 in a rather complementary fashion, with Nav1.1 expressed in the proximal part of the AIS, close to the soma, and Nav1.6 found more strongly expressed towards the distal AIS (Figure 3A-F). Analysis of Nav1.1 and Nav1.6 immunofluorescence intensity profiles along the AIS, as compared to that of AnkG, confirmed these two complementary distributions: intensity of Nav1.1 decreased when that of Nav1.6 increased (Figure 3F). In the remaining 20.2% of MNs, Nav1.1 was not expressed at the AIS and Nav1.6 was expressed along the entire AIS (Figure 3G-L), with an immunofluorescence intensity profile displaying a slightly lower level in the proximal AIS (Figure 3L).

We analyzed whether this new heterogeneity of AISs correlated with different functional types of MNs (α or γ) or with the first subdivision we observed into soma-derived or dendrite-derived MN AISs. We found that soma-derived AISs from α and γ MNs both display the two types of Nav composition in the same proportion (Table 1): about 80% of them express Nav1.1 and Nav1.6, while about 20% of them express only Nav1.6. As for dendrite-derived AISs (found only in 15% of α MNs), 100% of them express both Nav1.1 and Nav1.6 in two complementary AIS subcompartments (Table 1). Heterogeneous AISs in terms of localization and Nav channel composition can thus be found within a single neuronal population, and this heterogeneity differs among different functional types of MNs.

### Kv channel distribution in MN AISs

**Figure 4:**
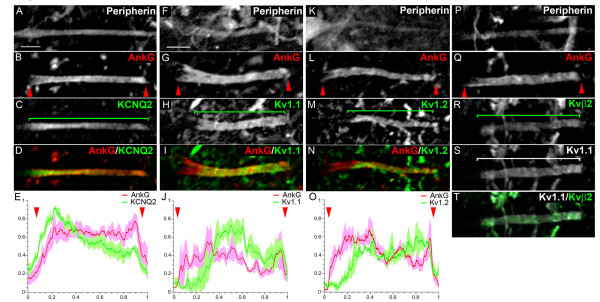
**Voltage-gated potassium (Kv) channel distribution in the axon initial segments (AISs) in motor neurons (MNs)**. Triple immunostaining of Peripherin **(A, F, K, P)**, ankyrin G (AnkG) **(B, G, L, Q) **and Kv channels: KCNQ2 **(C)**, Kv1.1 **(H, S)**, Kv1.2 **(M) **and Kvβ2 **(R) **(Kv and AnkG are merged in **(D, I, N)**; Kv1.1 and Kvβ2 are merged in **(T)**. **(E, J, O) **The mean immunofluorescence intensity profile (shown by the line) ± SEM from *n *= 6 AISs is shown for AnkG and Kv channels. For each AIS and each antibody, immunofluorescence intensities were normalized relative both to its maximum intensity along the AIS and to the length of the AIS. The beginning and the end of the AnkG^+ ^AIS in (B, G, L, Q) are shown (also in E, J, O) by red arrowheads. Brackets indicate Kv channel expression domains. Scale bar = 5 μm.

We next analyzed the MN AIS composition in terms of Kv channels. From this point, we focused our study on α MNs (identified either by their large soma or as NeuN^+^), because the following immunostainings were too weak in γ MNs to be properly analyzed. We found KCNQ2 expressed uniformly throughout the AnkG^+ ^AIS in 100% of α MNs (Figure 4A-E; *n *= 37): its immunofluorescence intensity profile along the AIS closely matched that of AnkG (Figure 4E). We also found Kv1.1, Kv1.2 as well as Kvβ2 expressed at the AIS of 100% of α MNs (respectively *n *= 214, *n *= 48 and *n *= 34), but all three, in contrast to KCNQ2, were clearly absent from the proximal AIS (Figure 4F-T), as depicted by their immunofluorescence profile (Figure 4J, O). The three subunits appeared perfectly colocalized as shown for instance for Kv1.1 and Kvβ2 (Figure 4R-T), suggesting that heteromultimeric channels formed by the association of Kv1.1, Kv1.2 and Kvβ2 are present in the AIS, as previously shown in juxtaparanodes (JXP-nodes) [36]. Unlike Nav1.6, which was always found expressed at the proximal AIS, even when Nav1.1 was present (although then at a lower level relative to the distal AIS: Figure 3D, F), Kv1.1, Kv1.2 and Kvβ2 channels appeared completely absent from the proximal AIS, displaying a clear change in their expression level between the proximal and distal AIS, as shown by their fluorescence intensity profile (Figure 4H, J, M, O). MN AISs can thus be divided into different subcompartments along their proximodistal length, which express various combinations of Nav and Kv channels.

### Identification of a 'para-AIS'

**Figure 5:**
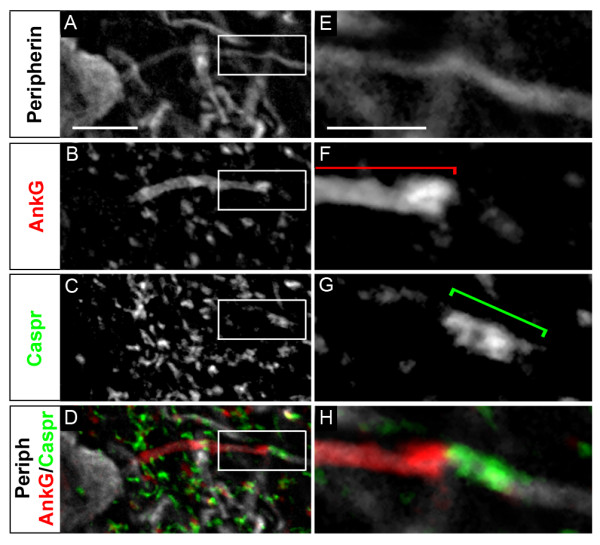
**Identification of a para-axon initial segment (AIS)**. Triple immunostaining of Peripherin **(A, E)**, ankyrin G (AnkG) **(B, F) **and contactin-associated protein (Caspr) **(C, G) **(merged in **(D, H)**) showing a Caspr-positive segment at the distal end of the AIS (D, H). (E-H) Magnified views of the frames in (A-D). Brackets indicate the AnkG^+ ^AIS and the Caspr^+ ^para-AIS. Scale bar = 10 μm (A-D), 2 μm (E-H).

Recent observations that some neurons could change their excitability properties as a mechanism of homeostatic plasticity, by either moving their AIS away from the soma [3] or increasing the length of their AIS [4], asks the question as to how far the AIS could respectively move or extend, or as a corollary where exactly beyond the AIS does the axon start being myelinated. Given the many similar features shared by AISs and nodes of Ranvier (including expression of AnkG, Nav and KCNQ2 channels), we analyzed whether AISs would, like nodes of Ranvier, be immediately followed by a myelinated axon and thus flanked by both a paranode-like and a contiguous JXP-node-like compartment. We therefore first analyzed the expression of the paranode marker, Caspr (also called Paranodin). Caspr is known to interact *in cis *with another paranodal cell-recognition protein, contactin, and their interaction allows the tight attachment of the myelin sheath paranodal loops to the axonal membrane. In 100% of somatic α MNs (*n *= 162), along their Peripherin^+ ^axon, we found Caspr expression directly contiguous to the AnkG^+ ^AIS (Figure 5A-H). This result suggests the presence of a paranode-like compartment immediately after the AIS. Given the differences between nodes of Ranvier and AISs, we refer here to this compartment as the 'para-AIS' (see Discussion).

### Identification of a 'juxtapara-AIS'

**Figure 6:**
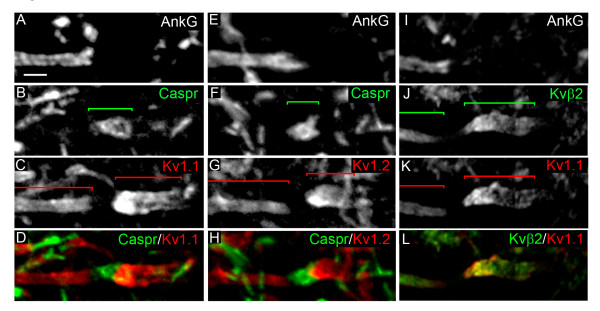
**Identification of a juxtapara (JXP)-axon initial segment (AIS)**. Triple immunostaining of ankyrin G (AnkG) **(A, E)**, contactin-associated protein (Caspr) **(B, F) **and voltage-gated potassium (Kv)1 channels **(C, G) **(Caspr and Kv1 are merged in **(D, H)**) along the axon of motor neurons (MNs) labeled with the anti-Peripherin antibody (data not shown). The Caspr^+ ^segment is immediately followed by a Kv1^+ ^segment: Kv1.1^+ ^(D) and Kv1.2^+ ^(H). Triple immunostaining of AnkG **(I)**, Kvβ2 **(J) **and Kv1.1 **(K) **(Kvβ2 and Kv1.1 are merged in **(L) **along the axon of MNs. Brackets indicate the Kv1^+ ^or Kvβ2^+ ^AIS and JXP-AIS and the Caspr^+ ^para-AIS. Scale bar = 5 μm.

We then analyzed whether the AIS and para-AIS were further flanked by a JXP-node-like compartment, which would further support the presence of a hemi-node-type organization at the distal tip of the AIS. We analyzed the expression of JXP-node markers, namely Kv1.1 and Kv1.2. In 100% of somatic α MNs (*n *= 68) we found Kv1.1 and Kv1.2 expressed in a compartment contiguous to the Caspr^+ ^para-AIS, on the opposite side of the distal AnkG^+ ^AIS expressing Kv1.1 and Kv1.2 (Figure 6A-H). This result suggests the presence of a JXP-node-like compartment, which we here refer to as a 'juxtapara-AIS' ('JXP-AIS'). Kvβ2, which we had found to be expressed in the distal AIS of MNs, was also expressed in their JXP-AIS (Figure 6I-L). As in the AIS, Kvβ2 was found perfectly colocalized with Kv1.1 (Figure 6J-L) and Kv1.2 (data not shown), suggesting that heteromultimeric channels formed by the association of Kv1.1, Kv1.2 and Kvβ2 are present in the JXP-AIS. Of note, their expression levels in the JXP-AIS, in particular in its proximal domain adjacent to the para-AIS, always appeared higher than in the AIS (Figure 6C, G, K). In addition, the axon displays a significant widening in the JXP-AIS as compared to the AIS, which contributes to the higher expression level of Kv1.1, Kv1.2 and Kvβ2 observed in the JXP-AIS.

In conclusion, all MNs have their AIS immediately flanked by a para-AIS and a JXP-AIS, which together strongly suggest the presence of a hemi-node type organization at the distal AIS and therefore the presence of a myelin sheath starting immediately after the AIS. However, given the differences between AISs and nodes of Ranvier, this hemi-node-type organization may display differences compared to a real hemi-node.

We investigated whether para-AISs and JXP-AISs can also be found in other neuronal types. In the cortex, we found, adjacent to the AIS, a Caspr^+ ^para-AIS and a contiguous Kv1.1^+ ^JXP-AIS in two neuronal types (see Additional file 1, Figure S1A-J), one of which being presumably a pyramidal cell (from the descending orientation and characteristic arrowhead-like morphology of its AIS: Additional file 1, Figure S1A-E). We also found a Caspr^+ ^para-AIS and a Kv1.1^+ ^JXP-AIS, adjacent to the AIS of Purkinje cells, which was surrounded by Kv1.1^+ ^basket cell terminals (Additional file 1, Figure S1K-O).

### Developmental expression of Kv1 channels at the MN AIS and JXP-AIS

**Figure 7:**
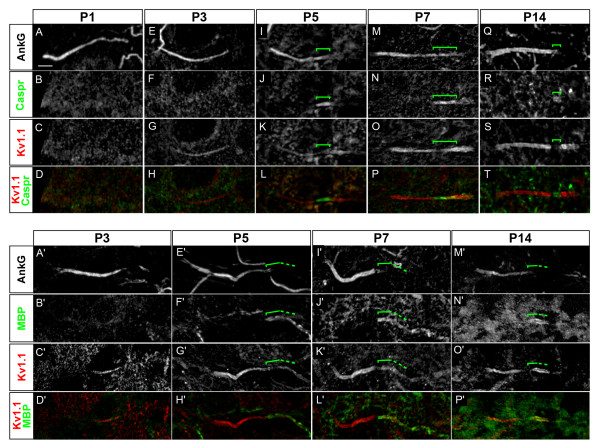
**Developmental expression of voltage-gated potassium (Kv)1 channels at the axon initial segment (AIS) and juxtapara (JXP)-AIS**. **(A-T) **Triple immunostaining of ankyrin G (AnkG) (A, E, I, M, Q), contactin-associated protein (Caspr) (B, F, J, N, R) and Kv1.1 channels (C, G, K, O, S) (Caspr and Kv1.1 are merged in D, H, L, P, T) along the axon of motor neurons (MNs) labeled with the anti-Peripherin antibody (data not shown), from P1 to P14. Brackets indicate the Caspr^+ ^domain (I-K, M-O, Q-S). **(A'-T') **Triple immunostaining of AnkG (A', E', I', M'), MBP (B', F', J', N') and Kv1.1 channels (C', G', K', O') (Caspr and MBP are merged in D', H', L', P') along the axon of MNs labeled with Peripherin (data not shown), from P3 to P14. Brackets indicate the MBP^+ ^domain (E'-F', I'-K', M'-O'). Scale bar = 5 μm.

Despite their crucial role in modulating the AIS spike-generating properties [12-14], the developmental time course of expression of Kv1 channels at the AIS has to date never been studied. Therefore, and as a first step towards investigating the mechanisms that could control the clustering of Kv1 channels at the AIS and JXP-AIS, we analyzed their developmental expression pattern in both compartments. We examined expression of Kv1.1 and Kv1.2 subunits in the ventral horn presumptive grey matter of mouse lumbar spinal cords at postnatal day 1 (P1), P3, P5, P7 and P14 (Figure 7A-T). We found an identical developmental distribution for Kv1.1 and Kv1.2 and thus restrict our following description to Kv1.1.

At P1, we did not find any expression of Kv1.1 in AnkG^+ ^AISs of Peripherin^+ ^MNs (*n *= 46) or in their presumptive JXP-AISs (Figure 7A-D). At P3, 83.3% of MNs (*n *= 38) were still not expressing Kv1.1 in their AIS or JXP-AIS, while 16.7% showed a weak expression of Kv1.1 in their AnkG^+ ^AIS, but did not yet express Caspr (as for the following stages/populations, a representative example is shown, in Figure 7E-H). At P3, expression of the myelin basic protein (MBP; a compact myelin marker) could not yet be detected (Figure 7A'-D'), which does not exclude the possibility that at this stage oligodendrocytes already start myelinating the axon of MNs at their distal AIS.

At P5, 67.3% of Peripherin^+ ^MNs (*n *= 36) were expressing Kv1.1. Strikingly, at this stage, Kv1.1 was never found expressed in the AIS or the presumptive JXP-AIS alone, but was always found expressed in both compartments together. Moreover, as soon as Kv1.1 could be detected at MN AISs, its distribution appeared restricted to the distal AIS (Figure 7G), as in adult MN AISs (Figure 4H), suggesting that the molecules responsible for segregating Kv1.1 within the AIS are already present and active at this early stage. Among the MNs expressing Kv1.1 at P5, 16.8% expressed Kv1.1 at a low level in both AIS and JXP-AIS, but did not yet express Caspr in their presumptive para-AIS; while 50.5% of MNs expressed Kv1.1 at a low to moderate level in both AIS and JXP-AIS and also expressed Caspr at the para-AIS (Figure 7I-L). Interestingly, at this early stage of Kv1.1 expression in AIS and JXP-AIS, the distribution of AnkG overlapped with that of Caspr (Figure 7I, J), suggesting that the AIS and para-AIS are not yet properly segregated. Similarly, the distribution of Kv1.1 was not well restricted to the AIS and the JXP-AIS and often extended into the para-AIS, where it overlapped with Caspr distribution (Figure 7J-L, 7N-P), resembling the initial stages of Kv1.1 expression in sciatic nerve JXP-nodes, where Kv1.1 extended into the Caspr^+ ^paranodal domain [37]. In addition, at P5, MNs that express Kv1.1 also display MBP staining along their axon, adjacent to the AIS (Figure 7E'-H'). This demonstrates the presence of a compact myelin sheath, which must be attached to the axon by adjacent paranodal loops (not labeled here by the anti-MBP antibody, as is often the case for paranodal loops) apposed to the adjacent Caspr^+ ^presumptive para-AIS. This result supports the previous conclusion that the para-AIS constitutes the first site of myelin attachment.

At P7, almost all MNs (92.2%, *n *= 37) now showed a significant expression level of Kv1.1 at both AIS and JXP-AIS, but AnkG and Kv1.1 were still overlapping with Caspr (Figure 7M-P). The remaining 7.8% of MNs expressed Kv1.1 at the AIS and JXP-AIS without Caspr. The segregation of the AIS, para-AIS and JXP-AIS into three mutually exclusive compartments occurred very progressively: at P14, 90% of MNs (*n *= 38) showed well segregated AnkG^+^, Caspr^+ ^and Kv1.1^+ ^compartments (Figure 7Q-T), similarly to Kv1.1 expression in sciatic nerve JXP-nodes, where it does not much extend any further within Caspr^+ ^paranodes [19,27]. MBP staining at P7 and P14 still reveals the presence of the myelin sheath adjacent to AIS (Figure 7I'-P').

### The TAG-1/Caspr2 complex is not required for Kv1 channels expression at the MN AIS and JXP-AIS in contrast to MN JXP-nodes

What controls the clustering of Kv1 channels at the AIS and JXP-AIS? Concerning the AIS and JXP-nodes, two different mechanisms have been proposed, respectively.

At the AIS, the membrane-associated guanylate kinase (MAGUK) PSD-93 was shown, using extinction with small hairpin (sh)RNAs in cultured hippocampal neurons, to control the clustering of Kv1 channels [15], even though analysis in *PSD-93*^-/- ^mice indicated that the lack of PSD-93 is compensated for [19]. At JXP-nodes, the clustering of Kv1 channels relies on two processes: it first requires an interaction with the JXP-nodal TAG-1/Caspr2 complex: in the absence of JXP-nodal Caspr2 (in Caspr2^-/- ^or *TAG-1*^-/- ^mice; [21]) or TAG-1 (in *TAG-1*^-/- ^mice; [20]), Kv1 channels failed to accumulate at JXP-nodes in sciatic and optic nerves. Second, paranodal axoglial junctions form a barrier that excludes Kv1 channels and restrict their clustering to JXP-nodes [24-27].

**Figure 8:**
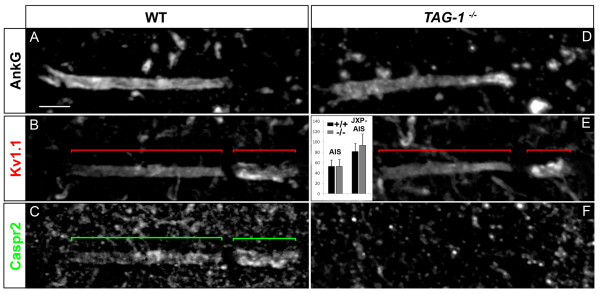
**The transient axonal glycoprotein-1 (TAG-1)/contactin-associated protein-like 2 (Caspr2) complex is not required for voltage-gated potassium (Kv)1 channels expression at the axon initial segment (AIS) and juxtapara (JXP)-AIS**. Triple immunostaining of ankyrin G (AnkG) **(A, D)**, Kv1.1 **(B, E) **and Caspr2 **(C, F) **in motor neurons (MNs) labeled with the anti-Peripherin antibody (data not shown), in wild-type (WT) (A-C) and *TAG-1^-/- ^*(D-F) mice. Inset in (E): Mean Kv1.1 immunofluorescence intensity at the AIS and JXP-AIS in WT and KO mice. Brackets indicate Kv1.1^+ ^and Caspr2^+ ^domains in the AIS and JXP-AIS. Scale bar = 5 μm.

We first tested whether the TAG-1/Caspr2 complex also controls the clustering of Kv1 channels at the MN JXP-AIS, similarly to JXP-nodes. Caspr2 is indeed a good candidate since it was distributed in the MN JXP-AIS, as well as in the MN AIS, exactly like Kv1 channels: in adult MNs its distribution was restricted to the distal AIS, overlapping exactly with Kv1.1 distribution, and was found at the JXP-AIS with a higher level of expression, similarly to Kv1.1 (Figure 8B, C). In addition, Caspr2 was also expressed at the MN JXP-AIS and AIS at the very early stage of Kv1 channels expression in these compartments (data not shown). We thus used *TAG-1*^-/- ^mice [38], in which Caspr2 expression was abolished at the MN JXP-AIS, as in JXP-nodes. Interestingly, Caspr2 expression was also missing at the MN AIS (Figure 8F). Surprisingly, in *TAG-1*^-/- ^mice we found a normal distribution of Kv1.1 in 100% of MN JXP-AISs analyzed, similar to wild-type (WT) littermate controls (Figure 8B, E): Kv1.1 at the MN JXP-AIS displayed no statistically significant difference of immunofluorescence intensity in *TAG-1*^-/- ^compared to WT mice (Figure 8E; from respectively 32 and 11 MN JXP-AISs analyzed from 3 *TAG-1*^-/- ^and 3 WT mice). This result contrasts with the dramatic decrease in Kv1 channels expression found at JXP-nodes in sciatic nerves [20,21], which include both motor and sensory axons. In order to test whether this discrepancy could be due to motor axons being less vulnerable to the lack of TAG-1 and Caspr2 than sensory axons, we analyzed more specifically teased fibers from spinal cord ventral roots contributing to the sciatic nerve, which contain JXP-nodes exclusively from MNs. In *TAG-1*^-/- ^mice, Kv1.1 expression was dramatically decreased in 88.1% of these MNs' JXP-nodes, as compared to WT mice (respectively 67 and 74 MN JXP-nodes analyzed from 3 *TAG-1*^-/- ^and 3 WT mice; see Additional file 2, Figure S2), like at sciatic nerve JXP-nodes [20,21]. Spinal somatic MNs thus have a JXP-AIS that differs from their peripheral JXP-nodes and from the majority of JXP-nodes studied so far, in that it seems to be able to cluster Kv1 channels by a TAG-1/Caspr2-independent mechanism.

With regard to the clustering of Kv1 channels at the MN AIS, expression of Kv1.1 in *TAG-1*^-/- ^mice displayed no statistically significant difference of immunofluorescence intensity compared to WT littermates (Figure 8B, E; respectively 32 and 15 MN AISs analyzed from 3 *TAG-1*^-/- ^and 3 WT mice), as was shown at the AIS of hippocampal and cortical neurons [15]. Thus, in contrast to JXP-nodes, the clustering of Kv1 channels at AISs, as at JXP-AISs, does not seem to require the TAG-1/Caspr2 complex in MNs.

### PSD-93 is not required for Kv1 channels expression at the MN AIS and JXP-AIS

**Figure 9:**
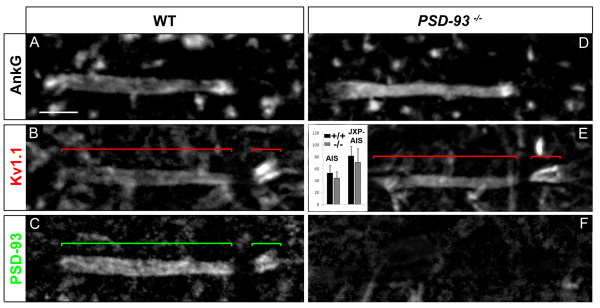
**PSD-93 is not required for voltage-gated potassium (Kv)1 channels expression at the axon initial segment (AIS) and juxtapara (JXP)-AIS**. Triple immunostaining of ankyrin G (AnkG) **(A, D)**, Kv1.1 **(B, E) **and PSD-93 **(C, F) **in motor neurons (MNs) labeled with the anti-Peripherin antibody (data not shown), in wild-type (WT) (A-C) and *PSD-93^-/- ^*(D-F) mice. Inset in (E): Mean Kv1.1 immunofluorescence intensity at the AIS and JXP-AIS in WT and KO mice. Brackets indicate Kv1.1^+ ^and PSD-93^+ ^domains in the AIS and JXP-AIS. Scale bar = 5 μm.

We then tested the second candidate, PSD-93, suspected to control or contribute to the clustering of Kv1 channels at the AIS [15,19] but not at JXP-nodes [39]. In line with the idea of PSD-93 playing a role in the clustering of Kv1 channels at the AIS, we found PSD-93 being restricted to the distal part of adult MN AISs, overlapping exactly with Kv1.1 distribution (Figure 9A-C). PSD-93 was also expressed at the MN AIS at the early stage of Kv1 channels expression at the AIS (data not shown). We thus analyzed *PSD-93*-null mice [40], in which we found a normal distribution of Kv1.1 in 100% of MN AISs, similar to WT littermate controls (Figure 9B, E): Kv1.1 at the MN distal AIS in *PSD-93*^-/- ^mice displayed no statistically significant difference of immunofluorescence intensity compared to WT mice (Figure 9E; respectively 14 and 11 MN AISs analyzed from 3 *PSD-93*^-/- ^and 3 WT mice). This result indicates that the clustering of Kv1 channels at the MN AIS does not require PSD-93.

Of note, we found PSD-93 being also expressed in the JXP-AIS, again matching exactly with Kv1.1 distribution (Figure 9B, C). In contrast to ventral roots contributing to the sciatic nerve, where PSD-93 was expressed in a minority of MN peripheral JXP-nodes (about 10% of them [39]; data not shown), we found PSD-93 expression in 100% of MN JXP-AISs (53 MN JXP-AISs analyzed), suggesting again that spinal somatic MNs have a JXP-AIS that differs from their peripheral JXP-nodes. This is further illustrated by the fact that the TAG-1/Caspr2 complex did not appear to control PSD-93 distribution in JXP-AISs (nor at the AIS; see Additional file 3, Figure S3), in contrast to sciatic nerve JXP-nodes [39] or more specifically to MN peripheral JXP-nodes (data not shown): PSD-93 expression was normal in all MN JXP-AISs analyzed in *TAG-1*^-/- ^mice (respectively 22 and 53 MN JXP-AISs analyzed from 3 *TAG-1*^-/- ^and 5 WT mice; Additional file 3, Figure S3). However, despite these differences between MN JXP-AISs and peripheral JXP-nodes, in both compartments PSD-93 did not appear to control the clustering of Kv1 channels. As in sciatic nerve JXP-nodes [39] and more specifically in MN peripheral JXP-nodes from teased ventral roots (respectively 123 and 74 MN JXP-nodes analyzed from 3 *PSD-93*^-/- ^and 3 WT mice; see Additional file 2, Figure S2), Kv1.1 distribution at MN JXP-AISs was not changed in *PSD-93*^-/- ^mice as compared to WT littermate controls (Figure 9B, E). Kv1.1 at the MN JXP-AIS in *PSD-93*^-/- ^mice displayed no statistically significant difference of immunofluorescence intensity compared to WT mice (Figure 9E; respectively 14 and 11 MN JXP-AISs analyzed from 3 *PSD-93*^-/- ^and 3 WT mice). Thus, the clustering of Kv1 channels in MN JXP-AISs, as in MN AISs, did not require PSD-93.

### Protein 4.1B is required to maintain a barrier at the MN para-AIS, necessary to cluster Kv1 channels and delimit the AIS, para-AIS and JXP-AIS

Finally, we tested the role of protein 4.1B in controlling both the hemi-node-like organization at the distal tip of the MN AIS and the clustering of Kv1 channels. 4.1 proteins are cytoskeletal proteins that play a key role in organizing membrane domains: they stabilize the membrane expression of a wide range of proteins by linking them to the actin/spectrin cytoskeleton [41]. Of the four members of the protein 4.1 family, protein 4.1B is expressed in myelinated axons at paranodes and JXP-nodes, where it binds Caspr and Caspr2, respectively [28,42]. Protein 4.1B has been shown to be necessary in JXP-nodes, for Caspr2 and thus Kv1 channels expression [29].

**Figure 10:**
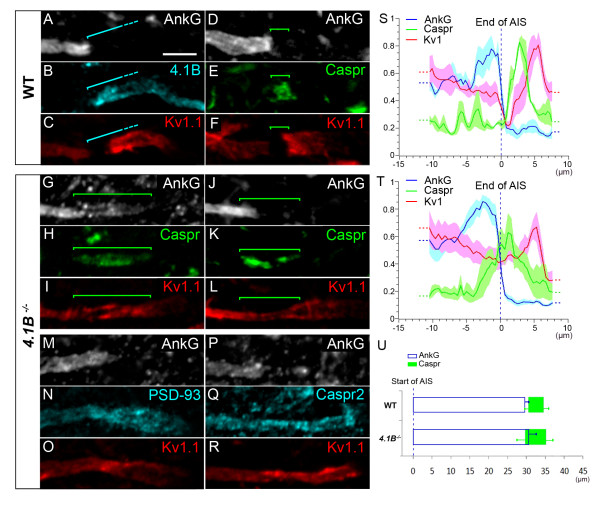
**Protein 4.1B is required to maintain a barrier at the para-axon initial segment (AIS) and to cluster voltage-gated potassium (Kv)1 channels**. Triple immunostaining of ankyrin G (AnkG) **(A)**, protein 4.1B **(B) **and Kv1.1 **(C) **in motor neurons (MNs) labeled with the anti-Peripherin antibody (data not shown) showing expression of protein 4.1B in the para-AIS, the juxtapara (JXP)-AIS and the internode. Triple immunostaining of AnkG **(D, G, J)**, contactin-associated protein (Caspr) **(E, H, K) **and Kv1.1 **(F, I, L) **in MNs of wild-type (WT) (D-F) and *4.1B^-/- ^*(G-L) mice showing an abnormal expression of Caspr and Kv1.1 in the para-AIS of *4.1B^-/- ^*mice. Triple immunostaining of AnkG **(M, P)**, PSD-93 **(N) **or Caspr2 **(Q)**, and Kv1.1 **(O, R) **in MNs of *4.1B*^-/- ^mice showing a similar abnormal expression of PSD-93, Caspr2 and Kv1.1 in the para-AIS. Brackets indicate protein 4.1B^+ ^(A-C) and Caspr^+ ^(D-L) domains. Scale bar = 5 μm. **(S, T) **The mean immunofluorescence intensity profile (shown by the line) ± SEM from *n *= 6 AISs is shown for AnkG, Caspr and Kv1.1 in WT (S) and *4.1B^-/- ^*mice (T). Axon segments were aligned at the end of their AIS (dashed line). For each axon segment and each antibody, immunofluorescence intensities were normalized relative to its maximum intensity along that segment. **(U) **Mean (± SEM) length of AnkG and mean (± SEM) beginning and end positions of Caspr from *n *= 11 (soma-derived) AISs, aligned at the beginning of their AIS (dashed line), from WT and *4.1B^-/- ^*mice.

We found protein 4.1B expression in MN axons, starting immediately after the AnkG^+^/Kv1^+ ^distal AIS, and covering the para-AIS, the Kv1^+ ^JXP-AIS, and extending beyond, within the internode (Figure 10A-F). Protein 4.1B could thus play an important role in stabilizing the molecular organization of the para-AIS and JXP-AIS. We therefore analyzed *4.1B*-null mice. We found that Caspr distribution was dramatically altered at the para-AIS of *4.1B*^-/- ^mice: it was not confined to a well delimited compartment between the AIS and the JXP-AIS, as in WT mice (Figure 10D-F); instead, it was extended along the axon, occupying either a single long Caspr^+ ^segment (Figure 10H) or dispersed Caspr^+ ^aggregates (Figure 10K; respectively 46 and 109 MNs analyzed from 3 *4.1B*^-/- ^and 12 WT mice), suggesting that the para-AIS was disrupted. In addition, Kv1.1 and AnkG distributions were not segregated from the Caspr^+ ^domain, as in WT mice (bracket, Figure 10D-F); instead AnkG (and Nav1.6) overlapped along varying distances with Caspr distribution (bracket, Figure 10G-H, 10J-K; see also Additional file 4, Figure S4A) and Kv1.1 distribution was continuous from the distal AIS to the JXP-AIS, covering completely the Caspr^+ ^area (bracket, Figure 10H-I, 10K, L). Analysis of AnkG, Caspr and Kv1.1 immunofluorescence intensity profiles from WT and *4.1B^-/- ^*AISs, aligned at the end of their AnkG staining, confirmed these observations (Figure 10S, T; *n *= 6 AISs from three WT mice and *n *= 6 AISs from three *4.1B^-/- ^*mice). These results suggest that protein 4.1B is required to maintain a normal distribution of Caspr and thereby an efficient barrier at the MN para-AIS: when disturbed, Kv1 channels invade the MN para-AIS. In order to analyze whether the overlap between Caspr and AnkG in *4.1B^-/- ^*mice corresponds to AnkG invading the para-AIS, or to Caspr invading the AIS, we analyzed the length of AnkG, and the position of Caspr relative to the beginning of the AIS (Figure 10U). The changes observed for the mean AIS length and the mean beginning of Caspr between WT and *4.1B^-/- ^*mice was not statistically significant (*n *= 9 AISs from three WT mice and *n *= 11 AISs from four *4.1B^-/- ^*mice). We can therefore only conclude that in *4.1B^-/- ^*mice AnkG and Caspr do not segregate properly from one another.

At the MN JXP-AIS, Kv1.1 expression was diminished in *4.1B*^-/- ^mice; its expression level in the JXP-AIS was not any higher than in the distal AIS, as in WT mice (Figure 10C, F); instead, in most cases it became even lower (Figure 10I, L, O). PSD-93 and Caspr2 were found to display the same distribution as Kv1.1: in contrast to WT mice (Figures 9C and 8C, respectively), PSD-93 and Caspr2 expression in *4.1B*^-/- ^mice also invaded the MN para-AIS and displayed a continuous distribution from the distal AIS to the JXP-AIS (Figure 10M-O and 10P-R, respectively).

## Discussion

### Heterogeneous AISs within the single neuronal population of MNs

The spiking properties of a neuron depend on its AIS ion channel composition, length and distance from the soma [1-4]. We have shown that within a single neuronal population, the somatic lumbar MNs, AISs can be heterogeneous both in terms of ion channel composition and localization, suggesting that the AIS may contribute to defining different functional cell types within a single neuronal population.

**Figure 11:**
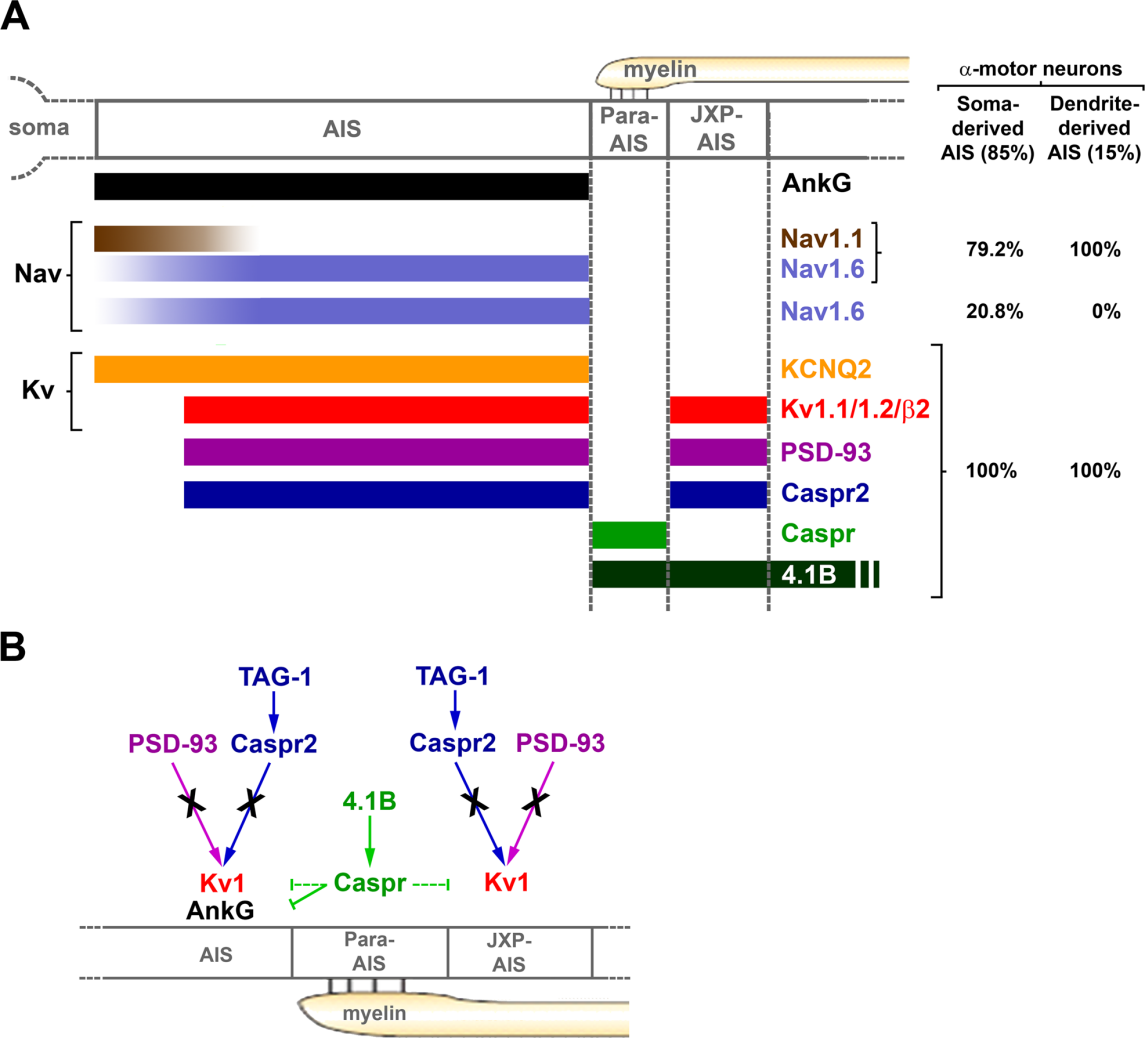
**Summary diagram**. **(A) **Localization and molecular composition of the axon initial segment (AIS), para-AIS and juxtapara (JXP)-AIS in α motor neurons (MNs). α MNs have either a soma-derived AIS (85% of α MNs) or a dendrite-derived AIS (15%). From the MNs with a soma-derived AIS, 79% of them express voltage-gated sodium channel (Nav)1.1 and Nav1.6, while 20.2% express Nav1.6 only. The remaining AIS, Para-AIS and JXP-AIS proteins were found in 100% of α MNs: KCNQ2 throughout the AIS only, while voltage-gated potassium channel (Kv)1.1/Kv1.2/Kvβ2, PSD-93 and contactin-associated protein-like 2 (Caspr2) in the distal AIS and JXP-AIS, Caspr in the para-AIS, and protein 4.1B in the para-AIS, JXP-AIS and the internode. **(B) **Kv1 channels are expressed in the AIS and JXP-AIS by mechanisms independent of transient axonal glycoprotein-1 (TAG-1)/Caspr2 and PSD-93, while protein 4.1B was shown to control Caspr expression in the para-AIS and thus the proper exclusion of Kv1 channels from the para-AIS and the proper segregation of AnkG and Caspr from one another.

We indeed found that, in contrast to γ MNs which all have a soma-derived AIS, α MNs have either a soma-derived or a dendrite-derived AIS (Figure 11A). To our knowledge such dendrite-derived axons in MNs have only been alluded to previously [43], but never described and quantified. The dendritic origin of the axon, reported in several neuronal types, may have a functional impact on the neuron's integrative properties. The axon-bearing dendrite, not separated from the AIS by the soma, is privileged in its ability to influence action potential initiation and synapses impinging on its proximal part, between the soma and the AIS, may act as gating synapses regulating how inputs from the soma and other dendrites might influence the AIS [44-46]. We also found that the distance from the soma to the AIS differs among MNs, and is longer in average for dendrite-derived AISs, which are thus more isolated from somatodendritic synaptic inputs and, as a consequence, might be less excitable [2,3].

Finally, AISs of MNs also differ with respect to their Nav channel composition, expressing either Nav1.6 only, or both Nav1.1 and Nav1.6 (Figure 11A). Nav1.1 may fulfill a similar role as Nav1.2 in layer 5 pyramidal neurons: due to their lower activation threshold, distal AIS Nav1.6 channels initiate action potentials, while higher threshold proximal AIS Nav1.2 channels ensure their backpropagation towards the soma [47]. This scenario was based on a comparison between Nav1.2 and Nav1.6 activation thresholds in neurons [48]. Such a comparison between Nav1.1 and Nav1.6 would help supporting a similar scenario for MNs. The higher impedance mismatch, that action potentials backpropagating from dendrite-derived AISs might have to overcome due to the larger proximal dendritic compartment, could explain why such AISs in MNs always express Nav1.1 in their proximal part.

These heterogeneous AISs, in terms of localization and Nav channel composition, may thus confer different excitability properties and contribute to define subsets of MNs with different firing properties [49]. Since intracellular recordings of adult mouse spinal MNs can now be performed [50,51], it will be possible to analyze the functional impact of these variable AIS parameters.

Of note, we found some AIS parameters that were instead constant across MNs, and may provide MNs with a common set of firing properties. This is the case for Kv channels distribution, even though regulatory mechanisms (for example, phosphorylation status, binding to interacting partners and in particular homo/heteromultimerization, binding to β subunits) could bring about differences between MNs. First, expression of slow subthreshold M-type K^+ ^current-mediating KCNQ2 channels [52], along the entire AIS of all α MNs (Figure 11A), might serve to control both the resting membrane potential and the action potential threshold, as they do in CA1 hippocampal pyramidal neurons [53]. Second, *shaker*-like Kv1.1 and Kv1.2 channels as well as the auxiliary Kvβ2 subunit were found to be colocalized at the distal AIS in all α MNs (Figure 11A), suggesting that they coassemble into heteromultimeric channels, as in juxtaparanodes [36]. These AIS Kv1 channels may function in MNs, as in layer 5 pyramidal neurons, to regulate the duration of action potentials [12] or, as in neocortical fast-spiking γ-aminobutyric (GABA)ergic interneurons, to control the action potential threshold and thus the firing rate [14]. Finally, AIS length was also rather constant among MNs, yet slightly different from other neuronal types (data not shown), suggesting that mechanisms must exist to regulate AIS length in a neuronal type-dependent manner.

### Identification of a para-AIS and a juxtapara-AIS

We chose to use here the terms 'para-AIS' and 'JXP-AIS' (instead of 'first paranode' and 'first JXP-node' for instance) for several reasons: these compartments abut an AIS, which displays important differences compared to a node of Ranvier; in addition these differences may well cause the para-AIS and JXP-AIS to differ from paranodes and JXP-nodes. Despite similarities, AISs and nodes indeed differ in terms of molecular composition (for instance, Kv1 channels and their binding partners Caspr2, TAG-1, PSD-93 and A Disintegrin And Metalloproteinase 22 (ADAM22) are expressed in AISs only), which implies different clustering/stabilizing mechanism. In addition, in contrast to nodes, AISs develop long before myelination. Thus, the mechanisms that cluster axonal proteins at the para-AIS and JXP-AIS upon myelination, at the distal tip of the preorganized AIS, might well differ from those operating at paranodes and JXP-nodes. Our results with *TAG-1^-/- ^*mice may illustrate such a difference.

The site where the axon starts being myelinated is still an open question for many neuronal populations, including mouse MNs: some chicken spinal MNs have a myelin sheath starting immediately after the AIS [54], while cat visceral preganglionic MNs have a myelin sheath starting several hundred microns after the AIS [45]. Our findings show that the axon of mouse somatic α MNs starts being myelinated immediately after the AIS and displays a hemi-node-type organization contiguous to the AIS, with a para-AIS and a JXP-AIS. The presence of the myelin sheath immediately after the AIS will allow action potentials to start their high velocity propagation along the axon and synchronously along its collaterals right from their site of initiation. In addition, it might change the ability of the AIS to increase either its length or its distance from the soma as a way for the neuron to adapt its excitability in response to changes in neuronal activity [3,4]. If MNs are able to display such homeostatic plasticity mechanisms, these might take place more easily during a critical period or during developmental stages preceding myelination, when MNs might need to adjust their intrinsic excitability to their changing synaptic inputs. Finally, given the presence of the myelin sheath immediately after the AIS (and the expression of Kv1 channels at the JXP-AIS), it is conceivable that demyelinating conditions may affect AIS spiking properties.

### Mechanisms that control Kv1 channel distribution in the AIS and JXP-AIS

The homogeneous distribution of KCNQ2 channels along the entire AIS, matching that of AnkG, reflects their AnkG-dependent clustering [17]. The mechanisms that segregate Nav channels in the AIS proximal (Nav1.1) or distal (Nav1.6) subcompartment, in addition to their AnkG-mediated membrane clustering, are currently unknown. Nav auxiliary β subunits (β1-4), which regulate Nav channel cell surface expression, interact with cell adhesion molecules and components of the extracellular matrix [55], could be potential candidates.

As for Kv1.1, Kv1.2 and Kvβ2, what controls their restricted expression in the distal AIS and in the newly characterized JXP-AIS? In *TAG-1^-/- ^*mice, their normal expression in both compartments indicates that TAG-1/Caspr2-independent mechanisms can control the distribution of Kv1 channels in the AIS and JXP-AIS, in contrast to the commonly accepted mechanism operating in JXP-nodes. Is this difference due to the presence of PSD-93 in all AISs and JXP-AISs (in contrast to only 7% of JXP-nodes in MNs [39]), and the fact that in both compartments PSD-93 distribution is not controlled by TAG-1/Caspr2 either (in contrast to JXP-nodes [39])?

PSD-93 was indeed shown, with shRNAs, to control Kv1 channels in the AIS of cultured hippocampal neurons [15]. However, Kv1 channels distribution was normal in the AIS of *PSD-93^-/- ^*mice [19]. The normal distribution of Kv1 channels found also in the AIS and JXP-AIS of *PSD-93^-/- ^*mice leaves open the possibility that PSD-93 plays a role *in vivo*: its absence in KO mice may lead to compensatory mechanisms [56], possibly mediated by other MAGUKs.

These results together with those from *TAG-1^-/- ^*mice support the existence of multifactorial mechanisms that can compensate for the absence of either PSD-93 or TAG-1/Caspr2 in the AIS and JXP-AIS (Figure 11B), but also in some JXP-nodes, where Kv1 channels expression was normal in Caspr2^-/- ^mice [19,56]. In *Caspr2^-/- ^*and *TAG-1^-/- ^*mice, the normal distribution of protein 4.1B respectively in JXP-nodes [29] and JXP-AISs (data not shown) could stabilize the expression of scaffolding proteins containing a PDZ domain and a protein 4.1-binding motif. Such scaffolds could allow PDZ-mediated recruitment of Kv1 channels. MPP2 and MPP6, members of the Membrane Palmitoylated Protein (MPP) family of MAGUKs [57], which can also bind Caspr2 [39], could play such a role and compensate for the absence of TAG-1/Caspr2 or PSD-93. Nectin-like proteins (Necls), which can bind PDZ-containing proteins (including MPP members [58]) and 4.1 proteins [59] may also contribute to such compensatory mechanisms. In AISs, other proteins may fulfill a similar role as protein 4.1B, or other mechanisms might exist to recruit Kv1 channels.

### Protein 4.1B is necessary to maintain a barrier at the para-AIS that compartmentalizes Kv1 channels and delimits the AIS, para-AIS and JXP-AIS

Recruitment of Kv1 channels at the AIS appears concomitantly with myelination and the formation of axoglial contacts at the AIS distal tip, as revealed by Caspr expression. Myelination may thus trigger the recruitment of Kv1 channels at the AIS, which may in part serve to adapt spike initiation properties to the new saltatory propagation and concomitant changes in firing properties [60].

Clustering of Caspr in the presumptive para-AIS indicates the formation of a membrane barrier, which progressively excludes Kv1 channels from this compartment and restricts their distribution to the AIS and JXP-AIS. The stabilization of the Caspr-expressing barrier is thus crucial for controlling the final distribution of Kv1 channels. Protein 4.1B, by linking Caspr to the cytoskeleton in the para-AIS, may play such a crucial role (Figure 11B): in its absence, Caspr and Kv1 channels distribution recapitulates an early developmental stage (such as P7), when Caspr is not yet properly clustered and does not form a complete barrier able to exclude all (JXP-AIS and/or AIS) Kv1 channels from the presumptive para-AIS. In *4.1B*^-/- ^mice, some Kv1 channels are found in the JXP-AIS (probably resulting from a weak barrier function at the para-AIS), where they might still be stabilized by 4.1B-independent mechanisms, as in the AIS. These results differ from the normal appearance of Caspr^+ ^peripheral paranodes observed in other *4.1B*^-/- ^mice [29], and suggest that the lack of protein 4.1B in the para-AIS is not compensated for by another 4.1 protein able to maintain a normal Caspr distribution (two new studies have analyzed the role of protein 4.1B in paranodes: one was published while our manuscript was under review [61], the other one is in press [62]).

Of note, the protein 4.1B-mediated consolidation of the Caspr-expressing barrier at the para-AIS ensures the proper segregation of Caspr not only from Kv1 channels, but also from AnkG: in *4.1B^-/- ^*mice, AnkG overlaps with Caspr (as at P7). Protein 4.1B thus plays a key role in controlling the hemi-nodal organization at the AIS distal tip and ensures the proper delimitation of the three compartments, the AIS, para-AIS and JXP-AIS.

## Conclusions

AISs from α MNs are homogeneous in terms of Kv1 and Kv7 channels composition but are heterogeneous in terms of axonal versus dendritic origin and Nav channel composition (Figure 11A). These heterogeneities suggest an important role for the AIS in defining subsets of motor neurons with different spiking properties. Despite these heterogeneities, all α MNs have their AIS immediately followed by a Caspr^+ ^paranode-type and a Caspr2^+ ^and Kv1 channels^+ ^JXP-node-type compartment (Figure 11A), which underlie the beginning of the myelin sheath and which might limit plasticity of AIS length or distance from the soma. Differences are observed at this first myelin attachment site compared with a hemi-node of Ranvier: in particular, Kv1 channels appear to be clustered in the JXP-AIS and AIS by TAG-1/Caspr2-independent mechanisms, while the cytoskeletal linker, protein 4.1B plays a crucial role in controlling the molecular organization of this region (Figure 11B): it is necessary to form the Caspr-expressing barrier at the para-AIS and to ensure the proper compartmentalization of Kv1 channels and the segregation of the AIS, para-AIS and JXP-AIS.

## Methods

### Animals

OF1 adult and postnatal mice (obtained from Charles River; L'Arbresle, France) were housed under standard laboratory conditions. *PSD93*-null, *TAG-1*-null and *4.1B*-null mice have already been described [38,40,62]. All animal experiments were performed in compliance with European Community guiding principles on the care and use of animals (86/609/EEC, EC off. J. no. L358, 18 December 1986), the French decree no. 97/748 of October 19, 1987 (J. Off. République Française, 20 October 1987) and recommendations from the CNRS and Université Pierre et Marie Curie.

### Antibodies

The following antibodies were used: anti-Nav1.1 rabbit polyclonal (AB5204, Millipore; Molsheim, France); anti-Nav1.6 mouse monoclonal (clone K87A/10, UC Davis/NIH NeuroMab Facility; Davis, CA, USA); anti-Peripherin rabbit polyclonal (AB1530, Millipore); anti-Kv1.1 mouse monoclonal (clones K20/78 and K36/15, UC Davis/NIH NeuroMab Facility); anti-Kv1.2 mouse monoclonal (clone K14/16, UC Davis/NIH NeuroMab Facility); anti-Kvβ2 mouse monoclonal (clone K17/70, UC Davis/NIH NeuroMab Facility); anti-Caspr (Paranodin) mouse monoclonal (clone K65/35, UC Davis/NIH NeuroMab Facility); anti-Caspr2 rabbit polyclonal (C8737, Sigma-Aldrich; Lyon, France); anti-Chapsyn-110 (PSD-93) mouse monoclonal (clone N18/30, UC Davis/NIH NeuroMab Facility), anti-MAP2 mouse monoclonal (M4403, Sigma-Aldrich), anti-Errγ. Err3) mouse monoclonal (H6812, PPMX; Tokyo, Japan), anti-NeuN mouse monoclonal (MAB377, Millipore) and anti-MBP rat monoclonal (MAB386, Millipore) antibodies. We also generated anti-KCNQ2 guinea pig polyclonal antibodies directed against residues 16-37 and 398-420 of human KCNQ2 and an anti-AnkG guinea pig polyclonal antibody directed against residues 1, 633-1, 650 of human AnkG. The anti-protein 4.1B antibody was a generous gift from Dr Laurence Goutebroze (INSERM UMR-S 839; Paris, France).

Alexa 405/Alexa 488/Alexa 594-conjugated secondary antibodies (Invitrogen; Villebon sur Yvette, France) were used to detect rabbit polyclonal or mouse monoclonal primary antibodies. Alexa 594-conjugated or Cy5-conjugated secondary antibodies (Jackson ImmunoResearch; Newmarket, Suffolk, UK) were used to detect guinea pig polyclonal primary antibodies.

### Immunohistochemistry

Adult mice were deeply anesthetized and fixed by intracardiac perfusion with 20 ml of 1% paraformaldehyde (PFA) freshly prepared in phosphate-buffered saline (PBS, pH 7.4) at 4°C. Lumbar spinal cords with their dorsal and ventral roots and brains were then immediately dissected out, further immersion fixed for 1 h in 1% PFA at 4°C, rinsed in PBS and cryoprotected by overnight immersion in 20% sucrose in PBS at 4°C. Spinal cords and brains from P3 to P21 were directly dissected out from deeply anesthetized mice, immersion fixed for 1 h in 1% PFA at 4°C and then rinsed and cryoprotected as adult tissues. Tissue samples were then frozen in OCT medium (VWR; Fontenay-sous-bois, France) and 20 μm cryosections were collected onto Superfrost Plus slides (VWR), and stored at -20°C. Ventral root sciatic nerves from spinal L3-L6 segments were desheated and teased with fine forceps on Superfrost Plus slides, air dried for 2 h and also stored at -20°C. Both types of slides were first thawed at room temperature (RT), washed in Tris-buffered saline (TBS), and incubated for 1 h in a blocking solution (10% goat serum in TBS) with 0.4% Triton X-100. They were then incubated overnight at 4°C with primary antibodies, diluted in the blocking solution with 0.2% Triton X-100, washed in TBS and incubated again for 2 h with secondary antibodies, diluted in the blocking solution with 0.2% Triton X-100. After washing in TBS, slides were dried out and mounted in Mowiol medium (Merk; Lyon, France). When primary antibodies generated in the mouse were used, prior to the primary antibody incubation step, slides were incubated for 3 h in goat anti-mouse F(ab)'2 (Jackson ImmunoResearch; Newmarket, Suffolk, UK), diluted in the blocking solution with 0.2% Triton X-100 and washed in TBS. This supplementary step reduced non-specific fixation of anti-mouse secondary antibodies onto mouse tissue.

When two rabbit-generated primary antibodies were combined in the same experiment, these were used sequentially: a first immunohistochemistry experiment was performed with one of them and after the incubation with its secondary antibody and washes, slides were incubated for 3 h in goat anti-rabbit F(ab)'2 (Jackson ImmunoResearch), diluted in TBS. Slides were then washed in TBS, incubated overnight at 4°C with the second rabbit-generated primary antibody, and processed normally until the end of this second immunohistochemistry. An identical experiment with the two primary antibodies used in the opposite order was carried out to confirm the absence of crossreactivity.

Images were acquired using a fluorescence microscope equipped with an Apotome module (Axiovert 200 M, Zeiss; Le Pecq, France) and a 63 × objective with non-saturating exposure times. Images were processed with the NIH ImageJ software (Bethesda, MD, USA). AIS length was measured with the 'Simple neurite tracer' plugin. Each figure corresponds to a projection image from a z stack of 0.6 μm distant optical sections, limited to the object of interest. Immunofluorescence intensity profiles were obtained with ImageJ, from a thick line (almost as large as the axon) traced along the axon segment, on a stack-of-interest projected image. For each antibody staining, the background intensity was first subtracted, and immunofluorescence intensity values along each axon segment were then normalized relative to the maximum intensity found along that segment. For each antibody, the mean intensity ± SEM from several axon segments is shown. In Figures 3 and 4, given the variable length of AISs and the consequent difficulty to align immunostaining profiles from several AISs, the positions along each AIS were first normalized relative to the length of that AIS. For Figure 10S, T, given the additional variability in the length of para-AISs and JXP-AISs, profiles were instead aligned at the end of the AIS, defined as the position along the axon where half of the maximum drop of AnkG immunostaining intensity (over 2 μm) is reached. In Figure 10U, the start and end positions of both AnkG and Caspr were similarly defined as the positions where half the maximum change of the respective immunostaining (over 2 μm) is reached. As for the analysis of Kv1.1 immunofluorescence intensity at the AIS and juxtapara-AIS in WT, *TAG-1*^-/- ^and *PSD-93*^-/- ^mice, the mean fluorescence intensity was measured with ImageJ on a selection square adjusted to the surface of the distal AIS and of the juxtapara-AIS, respectively. WT and mutant tissue sections were collected together on the same slide, in order for them to be processed in the same immunostaining experiment, thus with identical immunostaining conditions, and were analyzed in identical acquisition conditions (with the same exposure time).

The number of AISs analyzed for each figure (as indicated in the Results section) corresponds to the total number of AISs obtained from at least five animals, each analyzed in at least six independent experiments. We tested statistical significance using a Student's unpaired t test, and values are presented as mean ± SEM. For the comparison of distances from the soma to the AIS between soma-derived and dendrite-derived AISs, as well as for the comparison of AIS length and Caspr position between WT and *4.1B^-/- ^*mice, a Wilcoxon/Mann-Whitney test was used.

## Authors' contributions

AD participated in designing investigations, performed the experiments and data analysis, prepared the figures, wrote the Methods section, discussed and revised the manuscript. FCh and MG generated protein *4.1B-*null mice. FCo conceived the study and designed investigations, discussed and revised the manuscript. MD conceived the study and designed investigations, participated in some experiments and data analysis, and wrote the manuscript. All authors read and approved the final manuscript.

## Supplementary Material

Additional file 1 **Para-axon initial segment (AIS) and juxtapara (JXP)-AIS are found in other neuronal types**. Triple immunostaining of ankyrin G (AnkG) (A, F, K), contactin-associated protein (Caspr) (B, G, L) and voltage-gated potassium channel (Kv)1.1 (C, H, M) (merged in D, E, I, J, N, O) along the axon of P21 cortical neurons (A-E and F-J) and of adult Purkinje cells (K-O). Red brackets (C, H) indicate the Kv1.1^+ ^AIS and JXP-AIS separated by the Caspr^+ ^para-AIS, shown by the green bracket (B, G). In Purkinje cells, only one red bracket shows the Kv1.1^+ ^JXP-AIS, contiguous to the Caspr^+ ^para-AIS (L); the anti-Kv1.1 antibody also labels pinceau synapses around the AIS. Click here for file

Additional file 2 **Expression of voltage-gated potassium (Kv)1 channels in juxtapara (JXP) nodes in wild-type (WT), transient axonal glycoprotein-1 (*TAG-1*)*^-/- ^*and *PSD-93^-/- ^*mice**. Triple immunostaining of ankyrin G (AnkG) (B, D, F), contactin-associated protein (Caspr) (A-F) and Kv1.1 channels (A-F) (merged in B, D, F) in peripheral JXP-nodes of motor neurons (MNs) in WT (A, B), *TAG-1^-/- ^*(C, D), and *PSD-93^-/- ^*(E, F) mice. In A, C, E, immunostainings of Kv1.1 and Caspr, from nodes of Ranvier shown in B, D, F, respectively, have been shifted along the vertical dashed lines, in order to better visualize each immunostaining independently. Scale bar = 5 μm. Click here for file

Additional file 3 **Expression of PSD-93 at the axon initial segment (AIS) and juxtapara (JXP)-AIS in transient axonal glycoprotein-1 (*TAG-1*)*^-/- ^*mice**. Triple immunostaining of ankyrin G (AnkG) (A), voltage-gated potassium channel (Kv)1.1 (B) and PSD-93 (C) in motor neurons (MNs), labeled with Peripherin (data not shown), of TAG-1^-/- ^mice. Scale bar = 5 μm. Click here for file

Additional file 4 **Expression of ankyrin G (AnkG), contactin-associated protein (Caspr) and voltage-gated sodium channel (Nav)1.6 in *4.1B^-/- ^*mice**. Triple immunostaining of AnkG (A), Caspr (B) and Nav1.6 (C) (Caspr and Nav1.6 are merged in D) along the axon of motor neurons (MNs) (labeled with the anti-Peripherin antibody; data not shown) in *4.1B^-/- ^*mice. Brackets indicate the Caspr^+ ^domain (A-C). Scale bar = 5 μm. Click here for file
