# Supplementary figures and images for: Characterization of the axon initial segment (AIS) of motor neurons and identification of a para-AIS and a juxtapara-AIS, organized by protein 4.1B
Source: BMC Biol. 2011 Sep 29;9:66. doi: 10.1186/1741-7007-9-66 (PMC3198992; doi:10.1186/1741-7007-9-66)

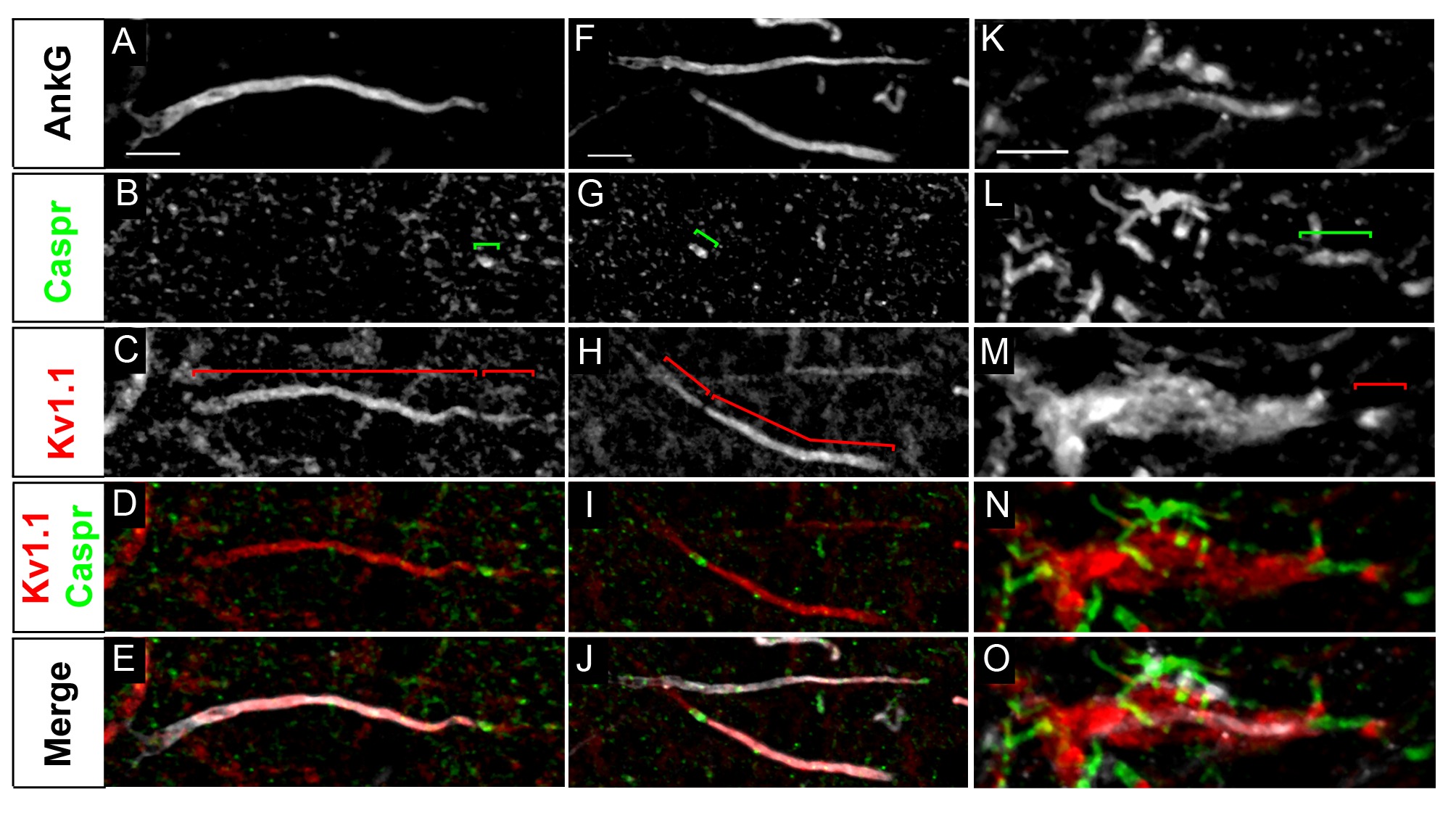

Supplement: Additional file 1 — Para-axon initial segment (AIS) and juxtapara (JXP)-AIS are found in other neuronal types. Triple immunostaining of ankyrin G (AnkG) (A, F, K), contactin-associated protein (Caspr) (B, G, L) and voltage-gated potassium channel (Kv)1.1 (C, H, M) (merged in D, E, I, J, N, O) along the axon of P21 cortical neurons (A-E and F-J) and of adult Purkinje cells (K-O). Red brackets (C, H) indicate the Kv1.1+ AIS and JXP-AIS separated by the Caspr+ para-AIS, shown by the green bracket (B, G). In Purkinje cells, only one red bracket shows the Kv1.1+ JXP-AIS, contiguous to the Caspr+ para-AIS (L); the anti-Kv1.1 antibody also labels pinceau synapses around the AIS. [file 1741-7007-9-66-S1.TIFF]

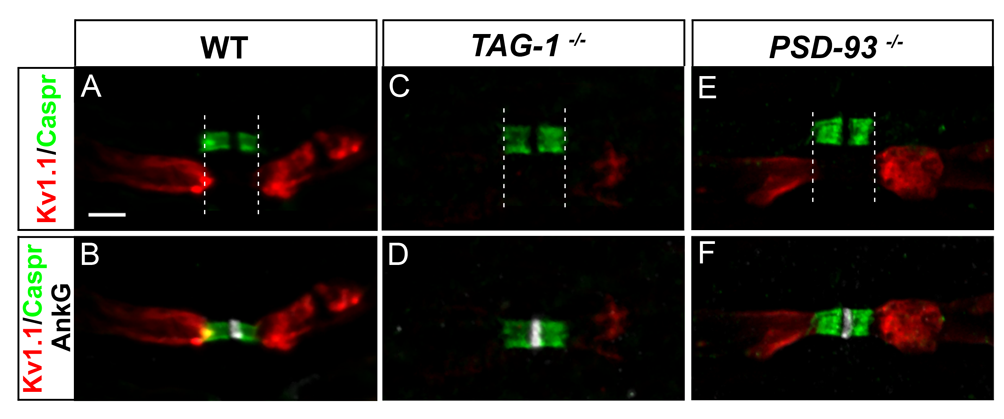

Supplement: Additional file 2 — Expression of voltage-gated potassium (Kv)1 channels in juxtapara (JXP) nodes in wild-type (WT), transient axonal glycoprotein-1 (TAG-1)-/- and PSD-93-/- mice. Triple immunostaining of ankyrin G (AnkG) (B, D, F), contactin-associated protein (Caspr) (A-F) and Kv1.1 channels (A-F) (merged in B, D, F) in peripheral JXP-nodes of motor neurons (MNs) in WT (A, B), TAG-1-/- (C, D), and PSD-93-/- (E, F) mice. In A, C, E, immunostainings of Kv1.1 and Caspr, from nodes of Ranvier shown in B, D, F, respectively, have been shifted along the vertical dashed lines, in order to better visualize each immunostaining independently. Scale bar = 5 μm. [file 1741-7007-9-66-S2.TIFF]

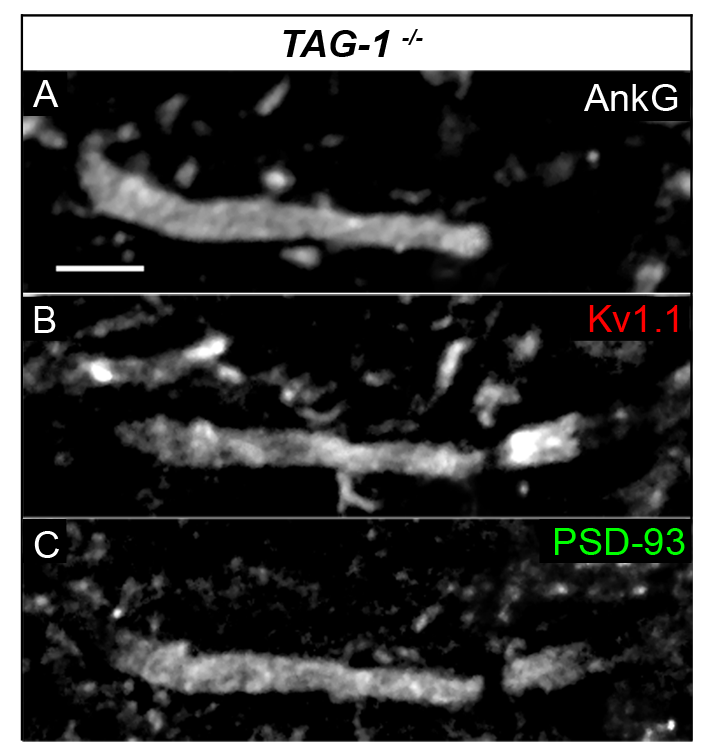

Supplement: Additional file 3 — Expression of PSD-93 at the axon initial segment (AIS) and juxtapara (JXP)-AIS in transient axonal glycoprotein-1 (TAG-1)-/- mice. Triple immunostaining of ankyrin G (AnkG) (A), voltage-gated potassium channel (Kv)1.1 (B) and PSD-93 (C) in motor neurons (MNs), labeled with Peripherin (data not shown), of TAG-1-/- mice. Scale bar = 5 μm. [file 1741-7007-9-66-S3.TIFF]

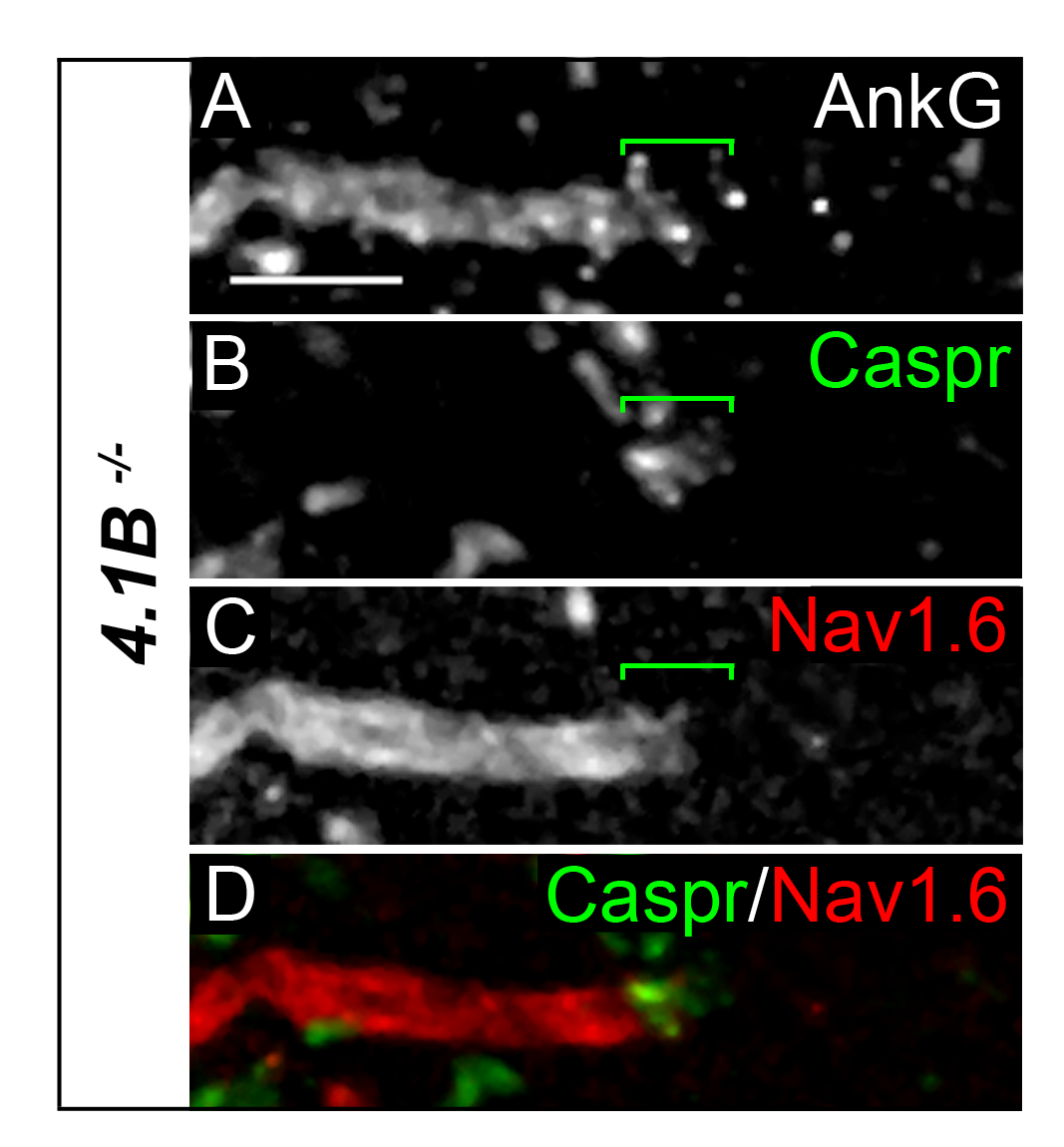

Supplement: Additional file 4 — Expression of ankyrin G (AnkG), contactin-associated protein (Caspr) and voltage-gated sodium channel (Nav)1.6 in 4.1B-/- mice. Triple immunostaining of AnkG (A), Caspr (B) and Nav1.6 (C) (Caspr and Nav1.6 are merged in D) along the axon of motor neurons (MNs) (labeled with the anti-Peripherin antibody; data not shown) in 4.1B-/- mice. Brackets indicate the Caspr+ domain (A-C). Scale bar = 5 μm. [file 1741-7007-9-66-S4.TIFF]
